# High-Dose Ethanol-Induced Immunosuppression Modulates Sex-Specific Disease Outcomes in a Murine Model of Multiple Sclerosis

**DOI:** 10.3390/biom16030427

**Published:** 2026-03-13

**Authors:** Adriana S. P. Nuncio, Katherine Motovilov, Max Weed, Simali Shah, Sam Bazzi, Esha Idnani, Turner Lime, Daniela Carrizales Sauceda, Regina A. Mangieri, Cole Maguire, Esther Melamed

**Affiliations:** 1 Department of Neurology, Dell Medical School, The University of Texas at Austin, Austin, TX 78712, USA; adrianaspn@utexas.edu (A.S.P.N.); katmotov@utexas.edu (K.M.); sam.bazzi@utsouthwestern.edu (S.B.);; 2 Department of Molecular Biosciences, The University of Texas at Austin, Austin, TX 78712, USA; 3 Department of Neuroscience, The University of Texas at Austin, Austin, TX 78712, USA; 4 University of Texas Southwestern (UTSW) Medical School, UTSW Medical Center, Dallas, TX 75390, USA; 5 Division of Pharmacology and Toxicology, College of Pharmacy, The University of Texas at Austin, Austin, TX 78712, USAdanielaguadalupe13@utexas.edu (D.C.S.); reginamangieri@utexas.edu (R.A.M.); 6 Department of Internal Medicine, Dell Medical School, The University of Texas at Austin, Austin, TX 78712, USA

**Keywords:** alcohol, multiple sclerosis, EAE, gut microbiome, immune system, sex differences

## Abstract

Both epidemiological studies and prior work in animal models suggest that moderate-dose alcohol reduces disease severity across several autoimmune conditions, including multiple sclerosis (MS). However, the mechanisms underlying the potentially beneficial effects of alcohol and how these effects may change with alcohol dose in autoimmunity remain underexplored. In this study, we characterize the effects of chronic, high-dose ethanol consumption in experimental autoimmune encephalomyelitis (EAE), a murine model of MS, by examining EAE disease severity, gut microbial composition, and peripheral cell immunophenotypes. We found that high-dose ethanol-fed males exhibited a significant amelioration in peak EAE disease severity, in association with decreased T cell activation and B cell proportions. Concurrently, we observed proinflammatory shifts in gut microbiota and hepatic lipid accumulation. Our results suggest that high dose ethanol may benefit autoimmune neuroinflammation in EAE through immunosuppressive effects on adaptive immunity, however its toxic systemic effects preclude the use of alcohol as an immunomodulator in MS. Overall, our findings reveal a mechanistic basis for alcohol’s beneficial properties in autoimmunity and could inform the development of more targeted disease modifying therapies that recapitulate these benefits without alcohol-associated toxicity.

## 1. Introduction

Multiple sclerosis (MS) is a demyelinating autoimmune disorder of the central nervous system (CNS) and is the main cause of non-traumatic neurological disability in young adults [1]. Recent evidence suggests that environmental factors, such as diet, play a prominent role in MS pathology, explaining up to 70% of MS risk [2,3]. One mechanism by which dietary factors are believed to influence MS is the gut microbiome, a dynamic network of bacteria, fungi, viruses, and other microorganisms essential in maintaining host homeostasis [4,5,6]. Through a two-way communication pathway between the gut and the CNS, known as the gut-brain axis, the gut microbiome is thought to influence the CNS and modulate the host’s immune system [5,6].

One common dietary factor is alcohol, with the prevalence of alcohol consumption in the MS patient population estimated to range from 13.6–40% [7,8]. Alcohol is known to exert diverse biological effects in a dose-dependent manner in the context of autoimmune disease [9,10,11,12] activating both pro- and anti-inflammatory pathways [13]. Importantly, patterns of alcohol use vary widely, ranging from low-to-moderate consumption to chronic high-dose intake characteristic of alcohol use disorder (AUD), a condition associated with sustained systemic and neuroimmune dysregulation [14,15,16].

In our previous work, we demonstrated that moderate-dose ethanol (2.6% alcohol-by-volume (ABV)) ameliorates autoimmune neuroinflammation in the experimental autoimmune encephalomyelitis (EAE) model in a sex-dependent manner, with males experiencing greater disease remission [17]. These effects were associated with alcohol-mediated increases in beneficial gut microbes, including *Akkermansia*, *Clostridium*, and *Prevotella* [17]. Similar findings have been reported in collagen-induced arthritis (CIA), a rodent model of rheumatoid arthritis (RA), where ethanol consumption reduced disease incidence and clinical severity [18]. The beneficial effects of ethanol on CIA were attributed, in part, to acetate-induced alterations in T follicular helper cell functional states, enhancing immune regulation [18]. Additionally, in epidemiological studies, low-to-moderate alcohol consumption has been associated with reduced risk, severity, or progression of several autoimmune conditions, including systemic lupus erythematosus (SLE), RA, autoimmune thyroid disease (AITD), and MS [19,20,21,22,23]. In contrast, chronic high-dose alcohol exposure, as observed in AUD, is frequently associated with neuroimmune activation, disruption of gut-brain homeostasis, systemic inflammation, and organ toxicity [13,24,25,26]. However, it is not well understood how alcohol at high doses impacts autoimmunity and whether it may be protective versus detrimental.

To address the gaps in our understanding of alcohol’s modulation of autoimmunity, we used the EAE model to investigate how chronic high-dose ethanol modulates autoimmune neuroinflammation. We assessed high-dose ethanol’s impact on disease severity, gut microbial composition, and immune cell phenotypes. Unexpectedly, our findings show that high-dose ethanol reduced EAE disease severity in a sex-dependent manner, concomitant with alterations in lymphocyte populations and the gut microbiome, revealing complex, multimodal effects on autoimmunity.

## 2. Materials and Methods

### 2.1. Animals and Ethanol Administration

C57BL/6J mice were purchased from The Jackson Laboratory (Bar Harbor, ME, USA) and group-housed in modified barrier cages (up to five mice per cage) under a 12 h light/dark cycle (total *n* = 92; ethanol-fed males: *n* = 25, ethanol-fed females: *n* = 25, control-fed males: *n* = 21, control-fed females: *n* = 21). All animal studies were approved by the University of Texas at Austin Institutional Animal Care and Use Committee (Animal Use Protocol 2023-00096). At 8 weeks of age, male and female mice were randomly assigned to one of two experimental groups: an ethanol-fed group receiving a Lieber–DeCarli ethanol liquid diet containing 5% alcohol-by-volume (ABV) (BioServ, Flemington, NJ, USA, F1259SP) or a control group receiving an ethanol-free isocaloric liquid diet (BioServ, Flemington, NJ, USA, F1258SP), with diet formulated as previously described [27]. Both liquid diets had a caloric density of approximately 1.0 kcal/mL. Mice were provided daily with liquid diet approximately six h into the light cycle to each cage using Bio-Serv feeding tubes, which were filled to the top (55 mL per cage), corresponding to a maximum caloric access of 55 kcal per cage per day, or 11 kcal per mouse per day. Water was provided ad libitum. The ethanol regimen included a 7-day ramp-up to 5% ABV: 1% (day 1), 2% (day 2), 2.6% (day 3), 3% (day 4), 4% (days 5–6), and 5% ABV (day 7), followed by 21 days of maintenance on the 5% ethanol diet prior to induction of experimental autoimmune encephalomyelitis (EAE) and continued throughout the duration of the experiment.

### 2.2. Blood Ethanol Concentrations

Blood samples were collected via terminal cardiac puncture approximately 2 h following initiation of the dark cycle at the cessation of the experiment for analysis of blood ethanol concentrations (BECs) (total *n* = 13; ethanol-fed males: *n* = 5, ethanol-fed females: *n* = 4, control-fed males: *n* = 2, control-fed females: *n* = 2). 5 µL of whole blood was added to 45 µL supersaturated sodium chloride inside of a 10 mL glass chromatography vial, and vials were immediately sealed by a screw cap. BECs were measured using flame-ionization detection gas chromatography (conditions described in Schier et al., 2012 [28]) with a Bruker 430-GC (Bruker Corporation, Fremont, CA, USA) equipped with CombiPAL SPME automated sample delivery system (Agilent Technologies, Cedar Creek, TX, USA) and analyzed with the Compass CDS Workstation software (Scion Instruments, Livingston, UK, GB-SCT). For this study, ethanol concentrations were determined using a six-point external standard curve (0–300 mg/dL).

### 2.3. Experimental Autoimmune Encephalomyelitis

EAE was induced in 12-week-old mice by subcutaneous injection of 200 µg of myelin oligodendrocyte glycoprotein peptide (MOG_35–55_; Genemed Synthesis Inc., San Antonio, TX, USA) emulsified in Complete Freund’s Adjuvant (CFA), prepared by suspending 200 mg of Mycobacterium tuberculosis H37Ra (BD Difco, Franklin Lakes, NJ, USA, #231141) in 25 mL of Incomplete Freund’s Adjuvant (IFA; BD Difco, Franklin Lakes, NJ, USA, #263910). Intraperitoneal injections of 110 ng of Pertussis toxin (PTX; lot #1016, Hooke Laboratories, Lawrence, MA, USA) were administered immediately following MOG_35–55_/CFA immunization and again 48 h post-immunization. Body weights and EAE scores were assessed daily using a 0–5 scale reflecting degree of limb paralysis and motor dysfunction according to the Hooke Laboratories protocol (“Appendix A: EAE scoring guide,” Hooke catalog no. EK-2110) [29]. Scores were assigned daily as follows: 0, no detectable clinical signs; 0.5, distal tail weakness; 1, complete tail paralysis; 1.5, tail paralysis with mild hind limb impairment; 2, tail paralysis with hind limb weakness; 2.5, tail paralysis with hind limb dragging; 3, tail paralysis with complete hind limb paralysis; 3.5, complete hind limb paralysis with inability to right when placed laterally; 4, complete hind limb paralysis with partial forelimb weakness; 4.5, severe paralysis with minimal spontaneous movement and reduced alertness; and 5, moribund or death. In this study, mice were evaluated across two cohorts. In Cohort 1, 40 mice were induced with EAE and monitored until Day 19. Cohort 2 was comprised of 52 mice total, which were monitored through Day 27 post-induction.

### 2.4. Liver Histology

Liver specimens were collected from ethanol-fed and control-fed animals (total *n* = 11; control-fed males: *n* = 2, control-fed females: *n* = 2, ethanol-fed males: *n* = 4, ethanol-fed females: *n* = 3) 27 days post-EAE induction for histological analysis, after 48 days of ethanol-diet or isocaloric control treatment. Livers were immediately drop-fixed and stored in cold 4% paraformaldehyde (PFA) overnight, then transferred to cold 30% sucrose the next day and stored overnight. The following day liver specimens were embedded in optimal cutting temperature (OCT) gel and flash frozen with dry ice and 2-methylbutane. Frozen liver blocks were subsequently sectioned on a cryostat at 10 µm, and 3 serial sections per animal were collected for histology. For Oil Red O (ORO) staining, an ORO stock solution was prepared from saturated ORO (Sigma-Aldrich, St. Louis, MO, USA; O0625) diluted in 99% isopropanol 3:1, then diluted in distilled water 3:2 to prepare an ORO working solution and filtered prior to use. Three serial sections were collected randomly every 10 µm for image acquisition and averaged as one mean value per animal. The liver sections were incubated in a working ORO solution and counterstained with 0.1% hematoxylin before mounting coverslips with PBS diluted in glycerol 9:1 and sealed with nail polish. Imaging was performed on a Nikon (Tokyo, Japan) Ni-E upright motorized microscope at 20× objective. Semi-quantitative lipid droplet analysis was performed in batch measurements on the top 200 lipid droplets via an automated script utilized with ImageJ software (Java v1.8.0_322), as previously described [30].

### 2.5. Fecal Microbiome Sequencing

A total of 54 fecal samples were sequenced from 27 mice (total *n* = 27; ethanol-fed males: *n* = 8, ethanol-fed females: *n* = 9, control-fed males: *n* = 5, control-fed females: *n* = 5) at two time points: prior to EAE induction and Day 26 post-EAE (during EAE remission). Samples were immediately frozen on dry ice after collection and stored at −80 °C until processing. Bacterial genomic DNA was extracted using the QIAamp PowerFecal Pro DNA Kit (Qiagen, Hilden, Germany, #51804) following the manufacturer’s instructions for whole genome metagenomics. The genomic DNA was quantified using the Qubit 4.0 Fluorometer (ThermoFisher Scientific, Waltham, MA, USA).

Library preparation and sequencing was conducted at GENEWIZ, LLC./Azenta US, Inc. (South Plainfield, NJ, USA). The NEBNext Ultra II DNA Library Prep Kit for Illumina (New England Biolabs, Ipswich, MA, USA) was used for library preparation according to the manufacturer’s recommendations. Briefly, genomic DNA was fragmented by acoustic shearing with a Covaris LE220 instrument (Covaris, Woburn, MA, USA) and was subsequently cleaned up and end repaired. Adapters were then ligated after adenylation of the 3′ ends followed by enrichment by limited cycle PCR. DNA libraries were subsequently checked on the Agilent TapeStation (Agilent Technologies, Palo Alto, CA, USA) and quantified using Qubit 4.0 Fluorometer as well as by real time PCR (KAPA Biosystems, Wilmington, MA, USA).

The sequencing libraries were multiplexed and sequenced using a 2 × 150bp Paired End (PE) configuration on the Illumina NovaSeq instrument (Illumina, San Diego, CA, USA). Image analysis and base calling were conducted by the NovaSeq Control Software (NCS), with the raw sequence data (.bcl files) converted into fastq files and de-multiplexed by the Illumina bcl2fastq (v2.20) software. One mismatch was allowed for index sequence identification.

Sequencing data were analyzed using the BioBakery workflow [31]. Taxonomic profiles generated by MetaPhlAn4 were imported into R for downstream analyses [32].

### 2.6. Flow Cytometry

Spleens were collected from ethanol-fed and control-fed male mice (*n* = 16; ethanol-fed males: *n* = 8, control-fed males: *n* = 8) on Day 19 post-EAE induction for flow cytometry analysis. Mice were anesthetized with isoflurane and euthanized via thoracotomy before blood was collected via cardiac puncture and spleens were removed and placed in phosphate buffered saline (PBS) at 4 °C. Spleens were processed into single-cell suspensions by mechanically homogenizing the tissue and passing the homogenate through a 40 µm mesh screen. Red blood cells were lysed with 1 mL red blood cell lysis buffer (155 mM NH4Cl, 10 mM KHCO3, 0.1 mM EDTA) for 1 min. Samples of 1 × 10^6^ cells were transferred to 5 mL flow cytometry tubes and pelleted by centrifugation. For surface staining, 100 μL of cell surface marker antibody cocktail (Table S1) was added and samples were incubated for 30 min at 4 °C in darkness. Following three washes with flow wash buffer (PBS containing 2% FBS), cells were fixed (Cytofix/Cytoperm Fixation/Permeabilization Kit BD Biosciences, Franklin Lakes, NJ, USA, #554714) for 30 min at 4 °C in darkness. After two washes, cells were resuspended in flow wash buffer and analyzed on a Cytek Aurora spectral flow cytometer (UT Center for Biomedical Research Support Microscopy and Flow Cytometry Core, RRID:SCR_021756). Data analysis was performed using FlowJo v10.9.0 (BD Biosciences, Franklin Lakes, NJ, USA).

### 2.7. Statistical Analysis

Differences in EAE clinical scores and body weight were analyzed using Student’s t-tests, with data presented as mean ± standard error of the mean (SEM). Disease severity during the peak of EAE (days 11–17 post-induction) was quantified as the number of days with high clinical scores ≥ 2) per mouse. Poisson regression between treatment and sex as factors was used to estimate predicted means and 95% confidence intervals, and pairwise comparisons between treatment groups within each sex were performed using estimated marginal means. Blood ethanol concentrations (BECs) were compared between ethanol-fed and control-fed mice using the Wilcoxon rank-sum test. For liver histology, 3 serial sections were averaged per animal, and differences between groups were assessed using the Wilcoxon rank-sum test with false discovery rate (FDR) adjustment. Flow cytometry proportions exported from FlowJo v10.9.0 were also assessed using the Wilcoxon rank-sum test.

For microbiome analyses, relative abundances from MetaPhlAn profiles were aggregated at the phylum and genus levels. Alpha diversity was quantified using the Shannon index and compared between groups with Wilcoxon rank-sum tests and FDR adjustment. Beta diversity was assessed using Bray–Curtis dissimilarities to characterize community structure and visualized using Principal Coordinates Analysis (PCoA). Differences in gut microbial composition, including the *Firmicutes/Bacteroidota* (FB) and *Verrucomicrobiota/Firmicutes* (VF) ratios, were assessed using Kruskal–Wallis tests followed by pairwise Wilcoxon rank-sum tests with Benjamini–Hochberg FDR adjustment. Taxa-level relative abundances at Day 26 post-EAE were compared between treatment- and sex-defined groups using pairwise Wilcoxon rank-sum tests. All statistical analyses and data visualization were performed in R (v4.5.0) using phyloseq, tidyverse, vegan, rstatix, ggplot2, ggpubr, and ggbeeswarm.

## 3. Results

### 3.1. Experimental Design and Ethanol Feeding

**Figure 1:**
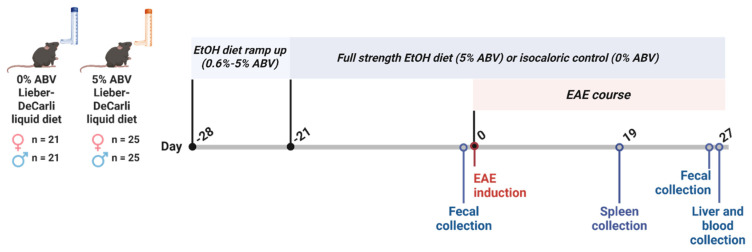
Experimental design. C57/BL6J male (♂) and female (♀) mice (*n* = 92) were fed a Lieber–DeCarli diet containing either ethanol (5% alcohol by volume, ABV) or an isocaloric control (0% ABV). Ethanol diet was increased from 1% to 5% ABV over 7 days and maintained throughout the study. Experimental autoimmune encephalomyelitis (EAE) was induced after 21 days of feeding at Day 0. Fecal samples were collected pre-induction and on Day 26 post-induction for whole genome metagenomics. Spleens (Day 19) and livers/blood (Day 27) were collected for immune profiling, histology, and blood ethanol concentration analysis.

Mice received a 5% alcohol-by-volume (ABV) Lieber–DeCarli diet or an isocaloric control diet starting 21 days before EAE induction and continuing through the end of the experiment. To evaluate the effects of high-dose ethanol administration at different stages of EAE, mice were euthanized at predefined time points: Day 19 post-EAE induction (following peak disease) for immune profiling and Day 27 (remission phase) for gut microbiome analysis, liver histology, and blood ethanol concentrations (BECs) (Figure 1).

### 3.2. High-Dose Ethanol Promotes Hepatic Lipid Accumulation

**Figure 2:**
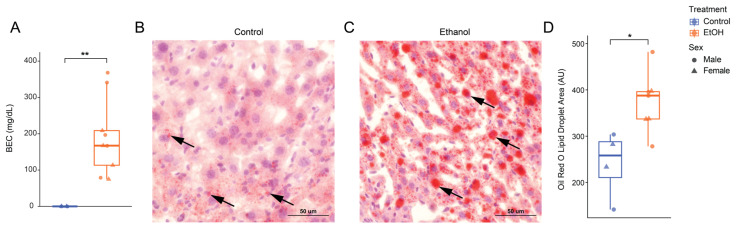
High-dose ethanol (5% ABV Lieber-deCarli, 48 d) is associated with hepatic lipid accumulation in mice. (**A**) Male and female mice receiving ethanol-diet reached significant blood ethanol concentrations (BECs), averaging ~170 mg/dL. (**B**,**C**) Representative Oil Red O (ORO)-stained liver sections from control- and ethanol-fed males taken at 20x with arrows indicating lipid droplets stained by ORO and (**D**) corresponding quantification of ORO lipid droplet area in males and females. Each point represents an individual mouse, with boxplots showing median and interquartile range. Alcohol-fed mice had significantly higher lipid accumulation compared with controls, as quantified by total lipid droplet area (Wilcoxon test, * *p* < 0.05, ** *p* < 0.01; total *n* = 11; control-fed males: *n* = 2, control-fed females: *n* = 2, ethanol-fed males: *n* = 4, and ethanol-fed females: *n* = 3). Images for quantification were acquired at 20x on a Nikon NiE Upright Brightfield microscope. Scale bar = 50 μm.

On average, ethanol-fed mice consumed approximately 15 g/kg of ethanol per day. As expected, blood ethanol concentrations (BECs) were significantly higher in ethanol-fed mice compared to controls, with the latter group having undetectable levels (control: 0 ± 0 mg/dL; ethanol: 191 ± 105 mg/dL; *p* = 0.0062; Figure 2A). Within the ethanol-fed group, BECs did not differ significantly between males and females (Table S2), indicating no sex-specific differences in circulating ethanol concentration.

To assess ethanol-induced hepatic lipid accumulation, we performed Oil Red O (ORO) staining on livers collected on Day 27 post-EAE induction [33,34], following 48 days of 5% ABV Lieber-DeCarli diet administration. Quantification of ORO-stained sections revealed that ethanol-fed mice exhibited significantly greater lipid accumulation compared with control-fed mice (Figure 2B–D). Control-fed mice displayed minimal lipid droplets, whereas ethanol-fed mice showed a 1.55-fold increase in lipid area (mean ± SD: Control = 240 ± 72 µm^2^, EtOH = 373 ± 64 µm^2^; *p* = 0.0297; Figure 2D), with no significant difference observed between male and female ethanol-fed mice (Table S3).

### 3.3. High-Dose Ethanol Ameliorates EAE in a Sex-Specific Pattern

Following EAE induction, mice were monitored daily for weight loss, symptom onset, and disease progression according to established EAE scoring criteria (“Appendix A: EAE scoring guide,” Hooke catalog no. EK-2110) [29,35]. A total of 92 mice were monitored until Day 19 post-EAE induction, while a subset of 52 mice continued to be monitored until Day 27. No significant differences in clinical scores were observed during the remission phase (Days 19–27) between experimental groups (Figure S1A). As expected, weight loss occurred at peak disease (Days 11–17 post-EAE induction) (Figure S1B).

**Figure 3:**
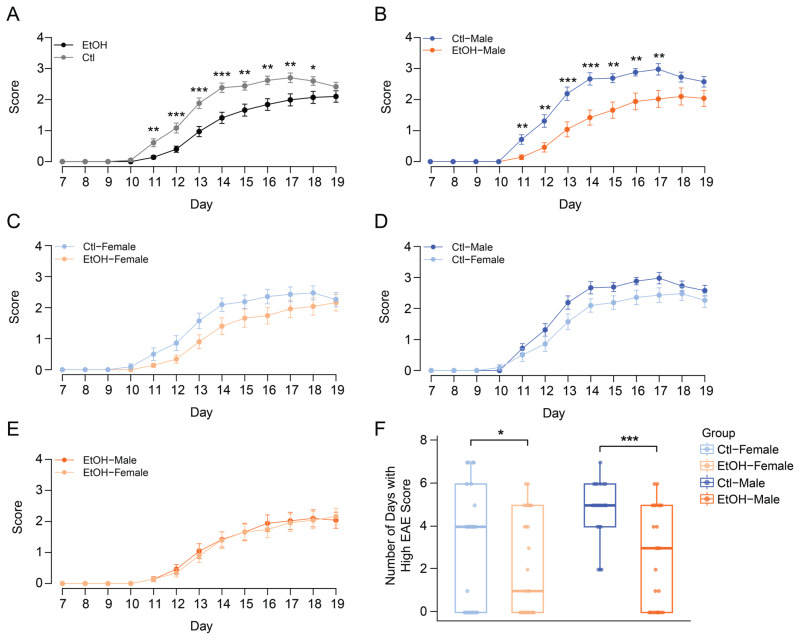
High-dose ethanol treatment ameliorates EAE course in a sex-specific pattern. (**A**) Animals receiving ethanol-diet showed significantly lower EAE scores from Days 11–18 compared to control groups within both sexes. (**B**) Ethanol-fed males showed significantly lower scores at disease onset compared to control-fed males. (**C**–**E**) No significant differences were observed between ethanol-fed females and control-fed females, control-fed males and females, ethanol-fed males and females (*n* = 21–25 per group; student’s *t* test, * *p* < 0.05, ** *p* < 0.01, *** *p* < 0.001). (**F**) Poisson regression of high-score days (EAE score ≥ 2, Days 11–17) revealed a significant treatment × sex interaction. Ethanol reduced high-score days in both sexes, with a stronger effect in males (* *p* < 0.05, ** *p* < 0.01, *** *p* < 0.001).

Motor deficits were first observed on Day 11, with peak disease scores occurring on Days 16 and 17 (*n* = 92, Figure 3A–E). When combining both sexes, ethanol-fed mice had significantly lower EAE scores compared to control-fed mice throughout the study (Figure 3A). Between Days 11–17, ethanol-fed males showed significantly lower scores at disease onset compared to control-fed males (Figure 3B). No significant differences were observed between ethanol-fed females and control-fed females (Figure 3C), control-fed males and control-fed females (Figure 3D), or ethanol-fed males and ethanol-fed females (Figure 3E) throughout the disease course.

Poisson regression model of disease severity was used to assess the number of days with high EAE scores (≥2) during the peak disease window (Days 11–17 post-induction). Ethanol-fed males exhibited significantly fewer high-score days (predicted mean ± 95% CI: 2.52 [1.97–3.23]) compared with control-fed males (4.81 [3.96–5.85], *p.adj* = 0.000057) (Figure 3F). Ethanol-fed females also had fewer high-score days (2.24 [1.72–2.91]) than control-fed females (3.33 [2.64–4.21], *p.adj* = 0.027), although the difference was smaller (Table S4). These results indicate that high-dose ethanol treatment reduced disease severity in both sexes, with a more pronounced effect in ethanol-fed males.

### 3.4. High-Dose Ethanol Alters Gut Microbial Composition Towards a Pro-Inflammatory Profile

**Figure 4:**
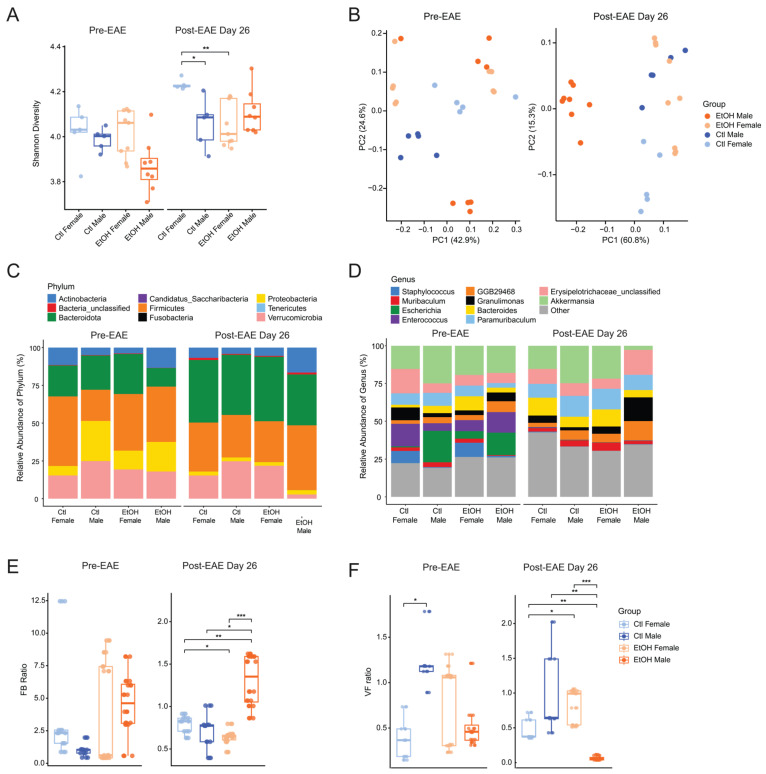
High-dose ethanol exposure and EAE alter gut microbiota alpha and beta diversity and relative taxa abundance in a sex-specific manner. (**A**) Alpha diversity (Shannon) of fecal microbiota at pre-EAE (left) and Day 26 post-EAE (right). Diversity was compared between groups using Wilcoxon rank-sum tests with FDR adjustment. (**B**) Beta diversity (Bray–Curtis) was visualized using Principal Coordinates Analysis (PCoA). Ethanol-fed males at Day 26 formed a distinct cluster, indicating differences in gut microbial composition. (**C**,**D**) Mosaic plots show relative abundances of gut microbiota at the phylum (**C**) and genus (**D**) levels across the four treatment groups. (**E**,**F**) *Firmicutes/Bacteroidota* (FB, (**E**)) and *Verrucomicrobiota/Firmicutes* (VF, (**F**)) ratios at pre-EAE induction (left) and Day 26 (right) post-EAE. Statistical differences were tested using Kruskal–Wallis with pairwise Wilcoxon tests and FDR adjustment (total *n* = 27; ethanol-fed females: *n* = 8, ethanol-fed males: *n* = 9, control-fed females: *n* = 5, and control-fed males: *n* = 5). * *p* < 0.05, ** *p* < 0.01, *** *p* < 0.001.

To determine whether high-dose ethanol consumption leads to alterations in gut microbial composition in the EAE model, we utilized whole genome metagenomics of fecal samples. Alpha diversity was quantified using the Shannon index and compared between groups using Wilcoxon rank-sum tests with FDR adjustment at pre-EAE and Day 26 post-EAE induction (EAE remission). At pre-EAE induction, Shannon diversity did not differ between groups (Figure 4A, left). By Day 26 post-EAE induction, control-fed females exhibited higher diversity than control-fed males (*p.adj* = 0.024) and ethanol-fed females (*p.adj* = 0.006), while other comparisons were not significant (Figure 4A, right). Principal-coordinate analysis (PCoA) of beta diversity, using the Bray–Curtis dissimilarity index, revealed distinct clustering based on both treatment and sex at pre-EAE induction and Day 26 (EAE remission) (Figure 4B). Notably, ethanol-fed males at Day 26 formed a separate cluster, distinct from the other groups (Figure 4B, right).

Analysis of gut microbiota relative abundance at both phylum and genus levels revealed sex differences among treatment groups, with a notable reduction in the *Verrucomicrobia* phylum (Figure 4C) and *Akkermansia* genus (Figure 4D) at Day 26 in ethanol-fed males, compared to all other treatment groups at both pre-EAE and Day 26 (Figure 4C,D).

Phylum-level microbiome ratios were assessed to evaluate changes in major bacterial groups (*Firmicutes* (F), *Bacteroidota* (B), and *Verrucomicrobiota* (V)) across treatment and sex prior to EAE induction and at Day 26 (EAE remission). Pre-EAE, no significant differences in the FB or VF ratios were observed across treatment or sex (Figure 4E,F, left). However, by Day 26, significant differences emerged. Ethanol-fed males exhibited a significantly higher FB ratio compared to control-fed males (*p.adj* = 0.025) and ethanol-fed females (*p.adj* = 0.003) (Figure 4E, right). In contrast, the VF ratio was decreased in ethanol-fed males relative to control-fed males (*p.adj* = 0.006) and ethanol-fed females (*p.adj* = 0.003) (Figure 4F, right). No significant differences were observed between control-fed males and females or between ethanol-fed and control-fed females. These findings indicate that high-dose ethanol selectively alters gut microbial composition in male mice during EAE remission, increasing the FB ratio while decreasing the VF ratio.

**Figure 5:**
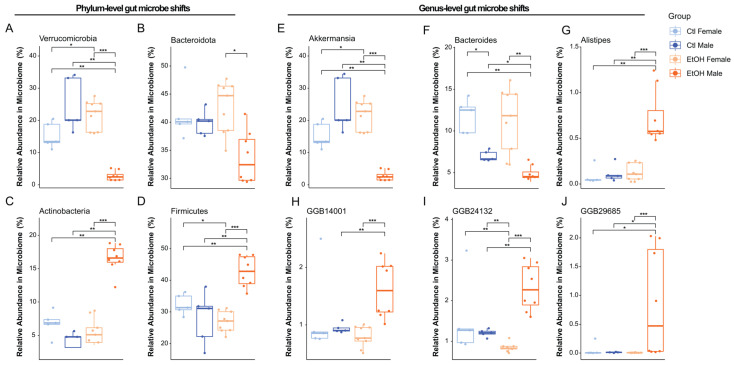
High-dose ethanol induces sex-specific shifts in key gut microbial taxa at the phylum and genus levels at Day 26 post-EAE induction. (**A**–**D**) Phylum-level abundances show that (**A**) *Verrucomicrobia* and (**B**) *Bacteroidota* are decreased, while *Actinobacteria* and (**D**) *Firmicutes* are increased in ethanol-fed males compared with other groups. (**E**–**J**) Genus-level abundances show reduced (**E**) *Akkermansia* and (**F**) *Bacteroides* and increased (**G**) *Alistipes* in ethanol-fed males. Uncultured taxa (**H**) GGB14001, (**I**) GGB24132, and (**J**) GGB29685 are also enriched in ethanol-fed males. Pairwise comparisons were performed using Wilcoxon rank-sum tests with FDR adjustment; * *p* < 0.05, ** *p* < 0.01, *** *p* < 0.001; individual points represent single mice, and boxplots show the median and interquartile range (total *n* = 27; ethanol-fed females: *n* = 8, ethanol-fed males: *n* = 9, control-fed females: *n* = 5, and control-fed males: *n* = 5).

Analysis of significantly altered taxa at Day 26 post-EAE induction revealed pronounced, sex-specific effects of ethanol treatment on gut microbial composition, particularly among ethanol-fed males (Figure 5). At the phylum level, *Verrucomicrobia* (Figure 5A) was significantly decreased in ethanol-fed males compared with control-fed males (*p.adj* = 0.0031) and ethanol-fed females (*p.adj* = 0.00049). Similarly, *Bacteroidota* (Figure 5B) abundance was reduced in ethanol-fed males relative to ethanol-fed females (*p.adj* = 0.015), though comparisons with control-fed groups did not reach FDR significance. In contrast, *Actinobacteria* (Figure 5C) and *Firmicutes* (Figure 5D) were significantly increased in ethanol-fed males compared with control-fed males (*p.adj* = 0.0031) and ethanol-fed females (*p.adj* = 0.00049).

At the genus level, *Akkermansia* (phylum *Verrucomicrobia)* was significantly decreased in ethanol-fed males compared to control-fed males (*p.adj* = 0.0031) and ethanol-fed females (*p.adj* = 0.00049) (Figure 5E). Similarly, *Bacteroides* (phylum *Bacteroidota*) abundance was significantly reduced in ethanol-fed males relative to control-fed males (*p.adj* = 0.012) and ethanol-fed females (*p.adj* = 0.0035) (Figure 5F). In contrast, *Alistipes* (phylum *Bacteroidota*) was significantly increased in ethanol-fed males compared to control-fed males (*p.adj* = 0.0031) and ethanol-fed females (*p.adj* = 0.00049) (Figure 5G). Additionally, uncultured taxa GGB14001 (phylum *Bacteroidota*) was significantly increased in ethanol-fed males compared with control-fed males (*p.adj* = 0.0093) and ethanol-fed females (*p.adj* = 0.00049). Similarly, GGB24132 (phylum *Bacteroidota*) was significantly increased in ethanol-fed males compared with control-fed males (*p.adj* = 0.0040) and ethanol-fed females (*p.adj* = 0.00049). Finally, GGB29685 (phylum *Firmicutes*) was significantly increased in ethanol-fed males compared with control-fed males (*p.adj* = 0.013) and ethanol-fed females (*p.adj* = 0.00049) (Figure 5H–J).

### 3.5. Ethanol-Treated Males Display Altered T and B Cell Proportions and T Cell Activation States

**Figure 6:**
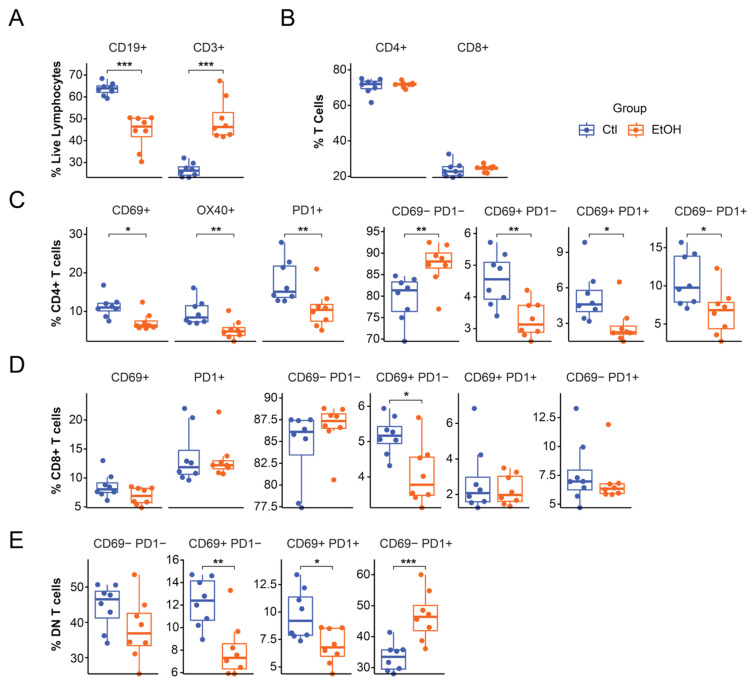
Males receiving high-dose ethanol demonstrate altered immune cell compositions, suggesting ethanol’s immunosuppressive effects. Percent of spleen lymphocytes which are (**A**) either CD19+ or CD3+ in control and ethanol-fed animals or (**B**) either CD4+ or CD8+ in control and ethanol-fed animals. Percent of spleen CD4+ T cells which are (**C**) positive for CD69, OX40, PD1 expression, or a combination phenotype of CD69 and PD1. (**D**) Percent of spleen CD8+ T cells which are positive for CD69, PD1, or a combination phenotype of CD69 and PD1 in control and ethanol-fed animals. (**E**) Percent of CD4- CD8- (double negative, DN) T cells which are positive for CD69 and/or PD1 expression in control and ethanol treated animals. Pairwise Wilcoxon rank-sum tests were used to evaluate significance; * *p* < 0.05, ** *p* < 0.01, *** *p* < 0.001 (total *n* = 16; ethanol-fed males: *n* = 8, control-fed males: *n* = 8).

To investigate whether ethanol-fed males experienced immunological changes corresponding with their improvement in disease course, we utilized immune cell phenotyping with flow cytometry, which revealed lymphocyte composition and activation in the spleen of animals receiving ethanol diet. Ethanol-fed males demonstrated a ~20% reduction in the proportion of CD19+ cells (B cells) (*p* = 0.0001) and a ~20% increase of CD3+ cells (T cells) compared to control-fed males (*p* = 0.00013) (Figure 6A). Interestingly, the relative proportions of CD4+ and CD8+ T cells were not altered by ethanol treatment (Figure 6B).

The proportion of CD4+ T cells displaying activation and/or exhaustion markers CD69, OX40, and PD1 was significantly reduced in ethanol-fed males (*p* = 0.015, 0.0081, and 0.0070, respectively) (Figure 6C). Examining co-expression of CD69 and PD1 (suggestive of differential activation state phenotypes) we found that the proportion of CD4+ T cells in activated states was significantly decreased in ethanol-fed males (CD69+PD1-, CD69+PD1+, CD69-PD1+; *p* = 0.0036, 0.023, 0.022 respectively) (Figure 6C). Simultaneously, the proportion of CD4+ T cells thought to be non-activated (CD69-PD1-) was significantly increased with ethanol-feeding *(p =* 0.0081) (Figure 6C). The same pattern was true for activation states marked by CD69/PD1 and OX40/PD1 co-expression in CD4+ T cells (Figure S2B).

CD8+ T cells did not demonstrate significant differences between groups in expression of CD69 or PD1 alone, however, the proportion of CD8+ T cells in the CD69+PD1- state was significantly decreased with ethanol feeding (*p* = 0.010) (Figure 6D). Finally, CD4-CD8- (double negative, DN) T cells in ethanol-fed males were found at lower proportions in the CD69+PD1- and CD69+PD1+ phenotypes and at higher proportions in the CD69-PD1+ phenotype (*p* = 0.00062, *p* = 0.012, *p* = 0.0015, respectively), with the proportion of naïve CD4-CD8- T cells (CD69-PD1-) trending down, as well (Figure 6E).

## 4. Discussion

In this manuscript, we present evidence that chronic exposure to high-dose ethanol, a dietary environmental factor commonly associated with immune activation and organ toxicity [14,15,16,36], can alter the course of autoimmune neuroinflammation. Using the experimental autoimmune encephalomyelitis (EAE) model of multiple sclerosis (MS), we demonstrate a sex-specific reduction in disease severity, most pronounced in male mice, which coincided with immunosuppressive modulation of the peripheral immune system. These immune alterations occurred alongside ethanol-induced inflammatory shifts in gut microbial composition and liver pathology, suggesting a duality in ethanol’s modulation of the gut-immune-CNS axis. Overall, our findings highlight alcohol as a complex modulator of autoimmune neuroinflammation and elucidate a potential mechanism by which alcohol, as a dietary factor, may shape disease course in MS.

Based on the prior evidence that alcohol promotes inflammation, oxidative stress, and hepatic injury [14,15,36,37,38], we anticipated that chronic high-dose ethanol would exacerbate EAE severity. Unexpectedly, we found that high-dose ethanol significantly reduced EAE disease severity, with the strongest effect observed in ethanol-fed males, extending our previous work demonstrating that moderate-dose ethanol ameliorates EAE in males [17]. Although high-dose ethanol attenuated EAE severity, our data also revealed hepatic lipid accumulation and gut dysbiosis in ethanol-fed animals. These findings suggest a dissociation between the neuroprotective effects of ethanol in autoimmunity and ethanol-induced peripheral organ damage, known to be widespread and affecting nearly all organ systems [15,36,39,40,41]. The adverse effects of alcohol emphasize that despite beneficial effects in autoimmunity, alcohol cannot be considered a direct therapeutic strategy for MS or other autoimmune conditions. However, our results provide a promising avenue towards elucidating the mechanisms underlying alcohol-induced immunomodulation.

One potential mechanism which may underlie alcohol-mediated autoimmune amelioration is the modulation of gut microbial communities [17]. In our study, high-dose ethanol altered gut microbial composition and diversity, with males showing the most pronounced beta-diversity shifts, suggesting that ethanol-feeding exerts a greater effect on the male microbiome. Ethanol-fed males further showed increased *Firmicutes/Bacteroidota* (FB) ratios, a widely used marker of gut microbial balance disrupted in alcohol use disorder (AUD) [42,43,44], driven by both elevated *Firmicutes* and reduced *Bacteroidota*, suggesting a shift towards a pro-inflammatory gut environment. Similarly, the *Verrucomicrobiota/Firmicutes* (VF) ratio, an indicator of mucin-degrading bacteria critical for gut barrier integrity [17], was reduced in ethanol-fed males, driven by depletion of *Akkermansia*, a genus central to epithelial barrier maintenance and immune regulation [45,46,47]. Lastly, our data demonstrate that ethanol-fed males showed reduced *Bacteroides* and increased *Alistipes*, another microbial signature linked to gut inflammation and previously observed in individuals with AUD [46]. This male-specific dysbiosis contrasts with moderate ethanol exposure, which we have previously found to increase *Akkermansia* and other anti-inflammatory taxa [17], highlighting a dose-dependent effect of alcohol on gut microbes. Additionally, we identified a novel association between high-dose ethanol and several unculturable genera within the *Firmicutes* and *Bacteroidota* phyla (GGB14001, GGB24132, GGB29685). While their functions remain unknown, their enrichment in phyla commonly associated with inflammation supports the idea that high-dose ethanol promotes potentially detrimental taxa capable of exacerbating gut dysfunction in males. Future studies could elucidate whether these genera play an essential role in driving inflammation or serve as passive biomarkers of gut dysbiosis. Collectively, our data indicate that high-dose ethanol promotes a male-biased, pro-inflammatory microbiome characterized by depletion of beneficial taxa and enrichment of potentially pathogenic genera.

Interestingly, despite proinflammatory microbial shifts, high dose ethanol-fed males exhibited EAE amelioration, suggesting benefits to neuroinflammation via an alternative mechanism, potentially of immune origin. Indeed, splenocyte immunophenotyping revealed that CD4+ (helper) T cells, and to a lesser extent CD8+ (cytotoxic) T cells, from ethanol-fed males displayed less activation and exhaustion markers during peak EAE disease. Our observed ethanol-linked shifts in CD4+ T cell activation and exhaustion may suggest that ethanol administration protects against CD4+ T cell overactivation in the setting of autoimmune disease. In support of this hypothesis, it has been previously shown that ethanol may decrease cytokine production in CD4+ T follicular helper cells, further indicating that ethanol exposure may mitigate inflammatory burnout of CD4+ T cells [18].

Notably, we found that CD4-CD8- (double negative, DN) T cells diverged from the pattern of CD4+/CD8+ activation/exhaustion phenotype proportions, instead demonstrating an increase in the proportion of CD69-PD1+ cells. DN T cells, including gamma-delta T cells or natural killer T (NKT) cells, are known to increase in autoimmune diseases and serve heterogeneous immunoregulatory functions, including playing a critical role in mucosal immunity [48,49]. In fact, small intestinal DN T cells have been previously shown to cross the epithelial barrier and tolerize naive CD4+ T cells in the lymph nodes, a mechanism by which they maintain mucosal homeostasis and prevent inflammation [50]. Thus, an increase in the fraction of DN T cells in an activation state may be a potential link between alcohol-mediated changes in gut microbial composition and alcohol’s immunomodulatory functions.

Both MS and EAE have been traditionally characterized as T cell driven conditions, where activated autoreactive CD4+ T cells infiltrate the CNS and trigger inflammation, demyelination, and neurodegeneration [51,52,53]. However, prior studies have demonstrated that B cells also contribute to MS and EAE disease course, directly activating pathogenic T cells [54,55,56,57]. Notably, B cell depleting disease modifying therapies (DMTs) have become a keystone MS treatment, showing significant benefits in reducing neuroinflammation, preventing relapses, and slowing disease progression [58,59,60,61]. In this manuscript, we observed that high-dose ethanol administration reduced the proportion of B cells in the spleen, which may, in part, explain our findings of EAE disease amelioration and draw a parallel to MS B cell depleting DMTs. A deeper examination in future studies of B cell phenotypes and how they may shift with ethanol administration in EAE and MS could contribute to the development of more specific next generation B cell depleting DMTs.

Interestingly, in the context of AUD, where individuals chronically consume alcohol at high doses, alcohol has also been shown to have immunosuppressive effects. Immunosuppression in AUD often manifests as increased susceptibility to infections and malignancies, as well as impaired responses to vaccinations [62,63]. These findings suggest that alcohol contributes to B cell dysfunction in AUD, similarly to the results in this study. Concurrently, epidemiological studies have shown that alcohol consumption may have a beneficial effect on disease severity in multiple autoimmune disorders including MS, rheumatoid arthritis, autoimmune thyroid disease, and systemic lupus erythematous [9,10,11,12]. These contrasting immune outcomes between AUD and autoimmune diseases suggest that alcohol-induced immunosuppression may serve as a double-edged sword, with effects heavily dependent on baseline immune state and disease context.

Previously, we have demonstrated that moderate-dose ethanol-feeding reveals a sex-specific effect, with males exhibiting greater benefit than females [17]. The results from this study reinforce that alcohol exerts sex-specific immunomodulatory and gut microbiome effects, which may be potentially mediated by differences in hormonal milieu, immune regulation and/or ethanol metabolism between males and females. Future studies should investigate the mechanisms driving these sex-specific effects to better inform how alcohol consumption may influence MS outcomes in men versus women.

The strengths of our study include a highly-powered design that interrogates the gut-immune-brain axis in a model of high-dose ethanol in EAE using whole genome metagenomics and broad cell immunophenotyping of the adaptive immune system. Our study also had limitations. First, we did not examine gut metabolites, which are known to be important mediators of gut-brain axis communication and future studies should interrogate their role in driving beneficial versus harmful effects with alcohol consumption in autoimmunity. Further, we investigated gut microbial composition only at two time points (prior to EAE induction and during EAE remission (Day 27)). Future studies should investigate microbiome changes longitudinally across EAE to better characterize dynamic microbial shifts during active inflammation and determine how microbiome alterations correlate with EAE disease progression and severity. Although we performed a broad evaluation of the immune system, we did not investigate B cell subsets or their activation states, which will be an important direction for further research to better ascertain the contribution of B cells to alcohol’s immunosuppressive effects in autoimmunity. Additionally, given the focus of our study on the adaptive immune system, we did not evaluate the innate immune system, which is also known to be influenced by alcohol [62,63]. While our analysis was limited to the spinal cord as the primary site of pathology in the C57BL/6J EAE model, future work examining alcohol’s impact on other CNS regions affected in MS would provide a more comprehensive understanding of alcohol’s immunomodulatory effects in the context of demyelinating disease. Lastly, our study was conducted in an animal model of MS receiving ethanol. Thus, future studies need to be translated to humans with MS and AUD given the important differences in disease pathology and ethanol metabolism between mice and humans.

## 5. Conclusions

In conclusion, our study provides the first mechanistic evidence that high dose alcohol ameliorates autoimmunity, most likely through immunomodulation of T and B cells. Concurrently, we confirm prior findings that high dose ethanol leads to gut dysbiosis and liver injury, emphasizing the fact that alcohol cannot be used as a treatment for autoimmune conditions. Nonetheless, the mechanistic understanding gained from this study paves the way towards the development of future DMTs in MS and other autoimmune diseases that may recapitulate alcohol’s underlying immunomodulatory mechanisms without the toxic, systemic effects of alcohol.

## Data Availability

The data reported in this paper are available via the Sequence Read Archive of the National Center for Biotechnology Information of the National Library of Medicine (SRA), published alongside this manuscript (BioProject ID: PRJNA1428757). All code is published at https://github.com/melamedlab/HighDoseEtOH_EAE (accessed on 2 February 2026).

## Supplementary Materials

The following supporting information can be downloaded at: https://www.mdpi.com/article/10.3390/biom16030427/s1, Table S1: Fluorescent antibodies used for immune cell phenotyping via flow cytometry and their proportions in the antibody cocktail used for staining; Table S2: Blood ethanol concentration (BEC) comparisons between groups using Wilcoxon rank sum test; Table S3: Oil O Red lipid droplet quantification comparisons between groups using Wilcoxon rank sum test; Table S4: Poisson regression model comparisons of high-score days (EAE score ≥ 2, Days 11–17); Figure S1: EAE scores and body weights. (A) EAE scores from the cohort of mice which were scored until Day 27 post-EAE induction (*n* = 52). Student’s *t* test, * *p* < 0.05, ** *p* < 0.01, *** *p* < 0.001). (B) Animal body weights, demonstrating expected decrease in body weight during peak EAE disease activity (Days 11–17); Figure S2: Flow cytometry data. (A) Flow cytometry gating strategy. (B) Percent of spleen CD4+ T cells positive or negative for activation/exhaustion markers, including CD69 and OX40 or OX40 and PD1 expression in control and ethanol treated animals. Pairwise Wilcoxon tests were used to evaluate statistical significance.
