# Supplementary material for: High-Dose Ethanol-Induced Immunosuppression Modulates Sex-Specific Disease Outcomes in a Murine Model of Multiple Sclerosis
Source: Biomolecules. 2026 Mar 13;16(3):427. doi: 10.3390/biom16030427 (PMC13023817; doi:10.3390/biom16030427)
Supplement: Supplementary file 1 [file biomolecules-16-00427-s001.zip › biomolecules-4161677-supplementary.pdf]

Supplementary Materials

Table S1. Fluorescent antibodies used for immune cell phenotyping via flow cytometry and their proportions in the antibody cocktail used for staining.

Target	Clone	Fluorophore	Manufacturer	Catalog number	Lot number	uL/test
CD19	6D5	BV750	BioLegend	115561	B459453	0.625
CD3	17A2	BV421	BioLegend	100228	B455718	1.25
CD4	GK1.5	PE	BioLegend	100407	B445229	1.25
CD69	H1.2F3	BV605	BioLegend	104530	B460580	2.5
CD8a	53-6.7	FITC	BioLegend	100706	B449123	2.0
CD134 (OX40)	OX-86	APC	BioLegend	119413	B473017	2.5
PD1	29F.1A12	PE-Cy7	BioLegend	135215	B474041	1.25
Viability		Zombie NIR	BioLegend	423105	B473817	1

Table S2. Blood ethanol concentration (BEC) comparisons between groups using Wilcoxon rank sum test.

Comparison	Test	P_value	Note
Ethanol-fed Male vs Female	Wilcoxon rank sum test	0.41	No significant difference between male and female ethanol-fed mice (Mean ± SD: Male 230 ± 122, Female 141 ± 59 mg/dL).
Control vs Ethanol (all sexes)	Wilcoxon rank sum test	0.0062	BECs are significantly higher in ethanol-fed mice compared to controls

Table S3. Oil O Red lipid droplet quantification comparisons between groups using Wilcoxon rank sum test.

Comparison	Test	P_value	Note
Control vs Ethanol (all sexes)	Wilcoxon rank-sum test	0.029	ORO significantly higher in ethanol-fed mice compared to controls
Ethanol-fed Male vs Female	Wilcoxon rank-sum test	0.860	No significant difference between male and female ethanol-fed mice

Table S4. Poisson regression model comparisons of high-score days (EAE score ≥ 2, Days 11–17).

contrast	EtOH / Ctl	EtOH / Ctl
Sex	Female	Male
ratio	0.672	0.52396
SE	0.120477	0.084118

 https://doi.org/10.3390/biom16030427

z.ratio	–2.21716	–4.02597
p.value	0.026612	5.67×10^{-5}
fold_change	0.67	0.52
perc_reduction	33	48

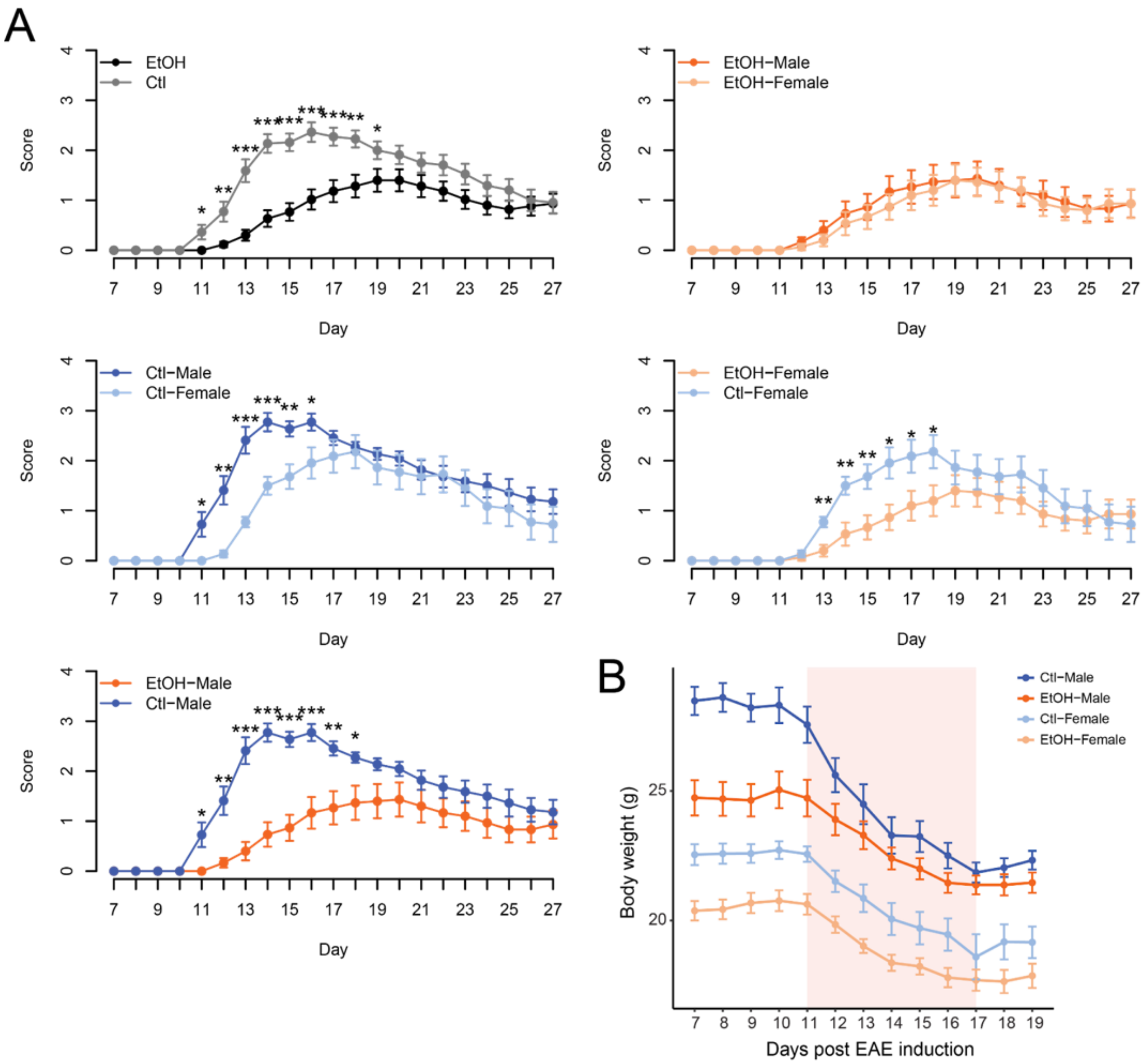

Figure S1. EAE scores and body weights. **(A)** EAE scores from the cohort of mice which were scored until Day 27 post-EAE induction (n = 52). Student's *t* test, *$p < 0.05$, **$p < 0.01$, ***$p < 0.001$). **(B)** Animal body weights, demonstrating expected decrease in body weight during peak EAE disease activity (Days 11–17).

https://doi.org/10.3390/biom16030427

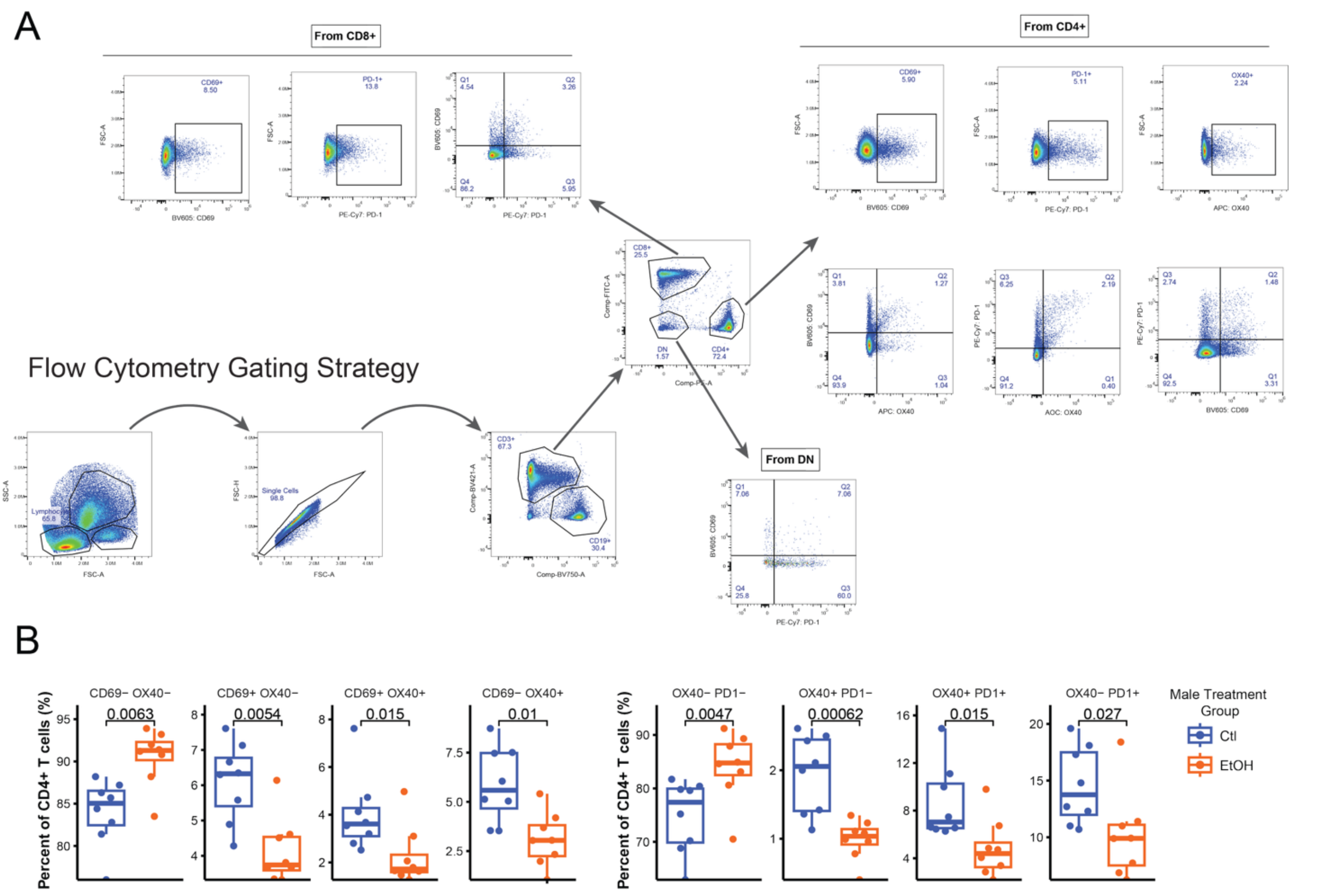

Figure S2. Flow cytometry data. **(A)** Flow cytometry gating strategy. **(B)** Percent of spleen CD4+ T cells positive or negative for activation/exhaustion markers, including CD69 and OX40 or OX40 and PD1 expression in control and ethanol treated animals. Pairwise Wilcoxon tests were used to evaluate statistical significance.
